# Supplementary material for: Potential of Urine Biomarkers CHI3L1, NGAL, TIMP-2, IGFBP7, and Combinations as Complementary Diagnostic Tools for Acute Kidney Injury after Pediatric Cardiac Surgery: A Prospective Cohort Study
Source: Diagnostics (Basel). 2023 Mar 9;13(6):1047. doi: 10.3390/diagnostics13061047 (PMC10047361; doi:10.3390/diagnostics13061047)
Supplement: Supplementary file 1 [file diagnostics-13-01047-s001.zip › Supplementary Figure S1.pdf]

Suppl. Figure S1: Schwartz estimate formula

GFR: glomerular filtration rate [18].

$$\boldsymbol{GFR}\ \left(\frac{\boldsymbol{ml}}{\boldsymbol{min}}\ \textbf{per 1.73 square m}\right) = \boldsymbol{k} * \frac{\boldsymbol{L}\ (\boldsymbol{cm})}{\boldsymbol{SCr}\ \left(\frac{\boldsymbol{mg}}{\boldsymbol{dl}}\right)}$$

$L = length$

$k = coefficient$

k = 0.33 in preterm infants (gestational age at birth < 38 weeks)
k = 0.45 in full-term infants
k = 0.55 in children (≥ 1 and < 13 years old) adolescent (≥ 13 y and < 21 y) girls
k = 0.70 in adolescent boys
