# Supplementary material for: Potential of Urine Biomarkers CHI3L1, NGAL, TIMP-2, IGFBP7, and Combinations as Complementary Diagnostic Tools for Acute Kidney Injury after Pediatric Cardiac Surgery: A Prospective Cohort Study
Source: Diagnostics (Basel). 2023 Mar 9;13(6):1047. doi: 10.3390/diagnostics13061047 (PMC10047361; doi:10.3390/diagnostics13061047)
Supplement: Supplementary file 1 [file diagnostics-13-01047-s001.zip › Supplementary tables S1 to S7.pdf]

Supplementary Table S1: Dilution of serum and urine samples for the initial measurement of CHI3L1 by ELISA

CHI3L1: chitinase 3-like protein 1; ELISA: enzyme-linked immunosorbent assay

Time point of the study	Estimated dilution for serum sample	Estimated dilution for urine sample
t0	1/10	1/2
t1	1/10	1/2
t2	1/20	1/2
t3	1/20	1/2
t4	1/20	1/2
t5	1/20	1/2
t6	1/20	1/2
t7	1/20	1/2

Supplementary Table S2: Adjustment of NGAL concentrations before input in statistical programs

LOD: limit of detection; LOQ: limit of quantification; NGAL: neutrophil gelatinase-associated lipocalin

Measured NGAL	Reported NGAL (ng/ml)
< LOD15	0.1
= LOD15	15.0
> LOD15 and < LOQ25	Median (LOD15, LOQ25) = 20.0
≥ LOQ25	Measured NGAL

Supplementary Table S3: Adjustment of urine IGFBP7 concentrations before input in statistical programs

AKIRisk: urine [TIMP-2]•[IGFBP7]; IGFBP7: insulin-like growth factor-binding protein 7; LL: lower limit of measurable range; TIMP-2: tissue inhibitor of metalloproteinases-2

Measured IGFBP7	Reported IGFBP7 (ng/ml)
< LL20	$\frac{[AKIRisk] * 1000}{[TIMP-2]}$

Supplementary Table S4: Additional pediatric patient health characteristics

ACE: angiotensin converting enzyme; AKI: acute kidney injury; CI: confidence interval; CRP: C-reactive protein; dCS: day of cardiac surgery; dPO1 postoperative day 1; h: hour; IQR: interquartile range; KDIGO: Kidney Disease Improving Global Outcomes; no: number; NSAID: nonsteroidal anti-inflammatory drug; PELOD: pediatric logistic organ dysfunction; SCr: serum creatinine, UO: urine output.

	**dCS**				**dPO1**				**dPO2**			
	**All patients (n = 101)**	**AKI stage ≥ 1[a] within 48-h (n = 63)**	**No AKI within 48-h (n = 38)**	**P value**	**All patients (n = 101)**	**AKI stage ≥ 1[a] within 48-h (n = 63)**	**No AKI within 48-h (n = 38)**	**P value**	**All patients (n = 101)**	**AKI stage ≥ 1[a] within 48-h (n = 63)**	**No AKI within 48-h (n = 38)**	**P value**
**Postoperative clinical examination (IQR)**												
PELOD score (n = 100, 99, 87)	5 (3-7)	5 (4-7)	4 (1-6)	0.002	2 (0-5)	4 (2-5)	1 (0-2)	< 0.001	1 (0-3)	2 (1-4)	0 (0-2)	< 0.001
Serum CRP – mg/l (n = 101, 101, 89)	2.2 (0.6-5.8)	2.3 (0.6-5.6)	1.9 (0.6-6.1)	0.961	23.9 (14.3-40.7)	22.5 (11.3-40.3)	27.8 (16.3-42.1)	0.398	36.0 (18.7-70.7)	36.1 (18.6-58.7)	35.7 (18.3-78.7)	0.750
Fluid balance – ml (n = 101, 101, 88)	167 (40-356)	170 (68-300)	165 (38-537)	0.697	171 (24-315)	171 (78-353)	164 (202neg-274)	0.202	47 (68neg-143)	66 (31neg-150)	1 (192neg-137)	0.041
**Postoperative transfusions – no. (%) [95 % CI]**												

Whole blood	20 (19.8) [13.2-28.6]	18 (28.6) [18.9-40.7]	2 (5.3) [1.5-17.3]	0.004	15 (14.9) [9.2-23.1]	13 (20.6) [12.5-32.2]	2 (5.3) [1.5-17.3]	0.044	8 (7.9) [4.1-14.9]	7 (11.1) [5.5-21.2]	1 (2.6) [0.5-13.5]	0.253
Plasma	40 (39.6) [30.6-49.4]	32 (50.8) [38.8-62.7]	8 (21.1) [11.1-36.3]	0.003	13 (12.9) [7.7-20.8]	11 (17.5) [10.0-28.6]	2 (5.3) [1.5-17.3]	0.123	2 (2.0) [0.5-6.9]	2 (3.2) [0.9-10.9]	0 (0.0) [0.0-9.2]	0.526
Platelets	4 (4.0) [1.6-9.7]	4 (6.3) [2.5-15.2]	0 (0.0) [0.0-9.2]	0.294	2 (2.0) [0.5-6.9]	2 (3.2) [0.9-10.9]	0 (0.0) [0.0-9.2]	0.526	3 (3.0) [1.0-8.4]	3 (4.8) [1.6-13.1]	0 (0.0) [0.0-9.2]	0.289
**Postoperative volumes transfused (IQR) – ml**												
Whole blood (n = 20, 15, 8)	43 (23-68)	48 (38-73)	20 (20-20)	0.063	40 (30-50)	40 (35-53)	35 (30-NA)	0.476	25 (20-78)	20 (20-90)	40 (40-40)	0.750
Plasma (n = 40, 13, 2)	50 (40-80)	55 (40-95)	45 (27-78)	0.415	60 (20-93)	20 (20-75)	94 (60-NA)	0.308	145 (50-NA)	145 (50-NA)	NA	NA
Platelets (n = 4, 2, 3)	55 (43-241)	55 (43-241)	NA	NA	188 (50-NA)	188 (50-NA)	NA	NA	140 (108-NA)	140 (108-NA)	NA	NA
**Postoperative antibiotics – no. (%) [95 % CI]**												
Any antibiotic	11 (10.9) [6.2-18.5]	8 (12.7) [6.6-23.1]	3 (7.9) [2.7-20.8]	0.528	27 (26.7) [19.1-36.1]	20 (31.7) [21.6-44.0]	7 (18.4) [9.2-33.4]	0.169	32 (31.7) [23.4-41.3]	25 (39.7) [28.5-52.0]	7 (18.4) [9.2-33.4]	0.029
Aminoglycosides	0 (0.0) [0.0-3.7]	0 (0.0) [0.0-5.7]	0 (0.0) [0.0-9.2]	NA	0 (0.0) [0.0-3.7]	0 (0.0) [0.0-5.7]	0 (0.0) [0.0-9.2]	NA	1 (1.0) [0.2-5.4]	1 (1.6) [0.3-8.5]	0 (0.0) [0.0-9.2]	1.000
β-lactams	10 (9.9) [5.5-17.3]	7 (11.1) [5.5-21.2]	3 (7.9) [2.7-20.8]	0.739	25 (24.8) [17.4-34.0]	19 (30.2) [20.2-42.4]	6 (15.8) [7.4-30.4]	0.153	30 (29.7) [21.7-39.2]	24 (38.1) [27.1-50.4]	6 (15.8) [7.4-30.4]	0.024
Glycopeptides	1 (1.0) [0.2-5.4]	1 (1.6) [0.3-8.5]	0 (0.0) [0.0-9.2]	1.000	2 (2.0) [0.5-6.9]	2 (3.2) [0.9-10.9]	0 (0.0) [0.0-9.2]	0.526	2 (2.0) [0.5-6.9]	2 (3.2) [0.9-10.9]	0 (0.0) [0.0-9.2]	0.526

Macrolides	0 (0.0) [0.0-3.7]	0 (0.0) [0.0-5.7]	0 (0.0) [0.0-9.2]	NA	0 (0.0) [0.0-3.7]	0 (0.0) [0.0-5.7]	0 (0.0) [0.0-9.2]	NA	1 (1.0) [0.2-5.4]	1 (1.6) [0.3-8.5]	0 (0.0) [0.0-9.2]	1.000
Chinolones	0 (0.0) [0.0-3.7]	0 (0.0) [0.0-5.7]	0 (0.0) [0.0-9.2]	NA	1 (1.0) [0.2-5.4]	0 (0.0) [0.0-5.7]	1 (2.6) [0.5-13.5]	0.376	1 (1.0) [0.2-5.4]	0 (0.0) [0.0-5.7]	1 (2.6) [0.5-13.5]	0.376
Co-trimoxazol	0 (0.0) [0.0-3.7]	0 (0.0) [0.0-5.7]	0 (0.0) [0.0-9.2]	NA	1 (1.0) [0.2-5.4]	0 (0.0) [0.0-5.7]	1 (2.6) [0.5-13.5]	0.376	0 (0.0) [0.0-3.7]	0 (0.0) [0.0-5.7]	0 (0.0) [0.0-9.2]	NA
**Other postoperative medication – no. (%) [95 % CI]**												
ACE inhibitors	1 (1.0) [0.2-5.4]	0 (0.0) [0.0-5.7]	1 (2.6) [0.5-13.5]	0.376	21 (20.8) [14.0-29.7]	16 (25.4) [16.3-37.3]	5 (13.2) [5.8-27.3]	0.206	21 (20.8) [14.0-29.7]	15 (23.8) [15.0-35.6]	6 (15.8) [7.4-30.4]	0.450
Diuretics	20 (19.8) [13.2-28.6]	11 (17.5) [10.0-28.6]	9 (23.7) [13.0-39.2]	0.453	70 (69.3) [59.7-77.5]	49 (77.8) [66.1-86.3]	21 (55.3) [39.7-69.9]	0.026	58 (57.4) [47.7-66.6]	43 (68.3) [56.0-78.4]	15 (39.5) [25.6-55.3]	0.007
Cyclosporine	1 (1.0) [0.2-5.4]	0 (0.0) [0.0-5.7]	1 (2.6) [0.5-13.5]	0.376	0 (0.0) [0.0-3.7]	0 (0.0) [0.0-5.7]	0 (0.0) [0.0-9.2]	NA	0 (0.0) [0.0-3.7]	0 (0.0) [0.0-5.7]	0 (0.0) [0.0-9.2]	NA
NSAIDs	46 (45.5) [36.2-55.2]	26 (41.3) [30.0-53.6]	20 (52.6) [37.3-67.5]	0.306	50 (49.5) [40.0-59.1]	30 (47.6) [35.8-59.7]	20 (52.6) [37.3-67.5]	0.684	43 (42.6) [33.4-52.3]	25 (39.7) [28.5-52.0]	18 (47.4) [32.5-62.7]	0.534
Corticosteroids	17 (16.8) [10.8-25.3]	11 (17.5) [10.0-28.6]	6 (15.8) [7.4-30.4]	1.000	17 (16.8) [10.8-25.3]	14 (22.2) [13.7-33.9]	3 (7.9) [2.7-20.8]	0.098	11 (10.9) [6.2-18.5]	8 (12.7) [6.6-23.1]	3 (7.9) [2.7-20.8]	0.528

Suppl. Table S5: Weight and length according to gender and age in AKI stage ≥ 1 versus no AKI patients within 48 hours after ICU admission

	all	AKI stage ≥ 1 within 48-h	No AKI within 48-h	P value
**Female**				
< 6m				
Length (cm)	62.0 [56.5-67.0]	62.0 [57.0-67.0]	60.5 [57.3-63.7]	0.582
Weight (kg)	5.2 [4.1-6.2]	5.2 [4.2-6.3]	5.1 [4.6-5.5]	0.727
≥ 6m & <3y				
Length (cm)	78.0 [65.0-85.0]	67.0 [65.0-78.0]	80.0 [78.0-82.0]	0.181
Weight (kg)	9.0 [6.6-10.2]	6.6 [5.8-9.6]	9.2 [9.0-10.4]	0.272
≥ 3y & <5y				
Length (cm)	90.0 [90.0-90.0]	-	90.0 [90.0-90.0]	
Weight (kg)	12.0 [12.0-12.0]	-	12.0 [12.0-12.0]	
≥ 5y & <10y				
Length (cm)	113.5 [106.5-121.03]	-	113.5 [106.5-121.03]	
Weight (kg)	19.6 [16.4-23.4]	-	19.6 [16.4-23.4]	
≥10 y&<18y				
Length (cm)	147.0 [145.5-154.0]	-	147.0 [145.5-154.0]	
Weight (kg)	37.0 [36.5-42.9]	-	37.0 [36.5-42.9]	
**male**				
< 6m				
Length (cm)	64.0 [62.5-68]	64.5 [61.1-68.5]	63.0 [63.0-63.0]	0.769
Weight (kg)	6.2 [4.5-7.7]	6.3 [4.5-7.7]	6.2 [6.2-6.2]	1.000
≥ 6m & <3y				
Length (cm)	74.0 [68.0-88.0]	70.0 [68.0-90.0]	78.0 [71.5-83.5]	1.000
Weight (kg)	8.6 [7.8-11.0]	8.0 [7.2-11.0]	9.3 [8.4-10.6]	0.730
≥ 3y & <5y				
Length (cm)	96.0 [94.5-100.0]	97.0 [96.0-103.0]	94.5 [93.8-95.3]	0.381
Weight (kg)	13.4 [13.0-15.1]	13.0 [12.9-15.5]	14.0 [13.7-14.3]	0.857
≥ 5y & <10y				
Length (cm)	104.0 [103.5-111.0]	104.0 [103.0-104.5]	129.0 [129.0-129.0]	0.500
Weight (kg)	15.6 [14.9-18.3]	15.2 [14.6-15.6]	25.0 [25.0-25.0]	0.500
≥10 y&<18y				

Length (cm)	166.0 [154.8-174.3]	173.5 [173.3-173.8]	157.5 [152.3-171.0]	0.643
Weight (kg)	56.7 [50.5-65.0]	61.5 [59.8-63.3]	54.3 [45.5-62.7]	0.429

Suppl. Table S6: Sensitivity analysis in pediatric patients without AKI ≥ 1 or AKI ≥ 2 on ICU admission for the respectively AKI prediction by biomarkers measured 4 hours after ICU admission in urine.

Data is presented as AUC-ROC values and 95% confidence interval. sCr: serum creatinine; CHI3L1: Chitinase-3-like protein 1; uCr: urine creatinine; NGAL: neutrophil gelatinase-associated lipocalin; TIMP-2: tissue inhibitor of metalloproteinases-2; IGFBP7: insulin-like growth factor-binding protein 7; NephroCheck® : AKI risk by analyzing [TIMP-2] and [IGFBP7]. Biomarkers/uCr: correction of the biomarker for urine dilution.

**outcome**	**AKI ≥ 1 within 48-h after ICU admission** AUC-ROC (95% CI)	**AKI ≥ 2 within 12-h after ICU admission** AUC-ROC (95% CI)	**P value** Compared to uCHI3L1/uCr For AKI ≥ 1 within 48-h after ICU admission	**P value** Compared to uCHI3L1/uCr For AKI ≥ 2 within 12-h after ICU admission
**outcome**				
CHI3L1	0.584 (0.453-0.707)	0.544 (0.435-0.650)		
CHI3L1/uCr	0.692 (0.561-0.804)	0.706 (0.598-0.799)		
NGAL	0.608 (0.477-0.729)	0.503 (0.395-0.612)		
NGAL/uCr	0.768 (0.642-0.866)	0.694 (0.585-0.789)	1.000	0.847
NephroCheck®	0.594 (0.447-0.729)	0.523 (0.399-0.645)		
NephroCheck® /uCr	0.705 (0.559-0.825)	0.760 (0.641-0.855)	0.492	0.128
TIMP-2	0.508 (0.364-0.651)	0.514 (0.390-0.636)		
TIMP-2/uCr	0.681 (0.534-0.805)	0.813 (0.700-0.897)	0.765	0.040
IGFBP7	0.650 (0.503-0.778)	0.589 (0.464-0.706)		
IGFBP7/uCr	0.776 (0.636-0.882)	0.820 (0.708-0.903)	0.067	0.001
ΔsCr[postop-preop]	0.869 (0.722-0.955)	0.866 (0.743-0.944)	0.027	0.001

Suppl. Table S7: Sensitivity analysis in pediatric patients without AKI ≥ 1 or AKI ≥ 2 on ICU admission for the respectively AKI≥ 1 and AKI≥ 2 prediction by biomarker combinations measured 4 hours after ICU admission in urine.

Data is presented as AUC-ROC values and 95% confidence interval. sCr: serum creatinine; CHI3L1: Chitinase-3-like protein 1; uCr: urine creatinine; NGAL: urine neutrophil gelatinase-associated lipocalin; TIMP-2: tissue inhibitor of metalloproteinases-2; IGFBP7: insulin-like growth factor-binding protein 7; NephroCheck® : AKI risk by analyzing [TIMP-2] and [IGFBP7].

**outcome**	**AKI ≥ 1 within 48-h after ICU admission** AUC-ROC (95% CI)	**AKI ≥ 2 within 12-h after ICU admission** AUC-ROC (95% CI)
**Combinations**		
**All corrected for uCr:**		
[CHI3L1]•[TIMP-2]	0.655 (0.504-0.786)	0.702 (0.574-0.810)
[CHI3L1]•[IGFBP7]	0.698 (0.549-0.822)	0.722 (0.596-0.827)
[CHI3L1]•[NephroCheck®]	0.685 (0.535-0.811)	0.720 (0.594-0.825)
[CHI3L1]•[NGAL]	0.698 (0.564-0.812)	0.654 (0.540-0.756)
[NGAL]•[TIMP-2]	0.706 (0.557-0.828)	0.683 (0.554-0.793)
[NGAL]•[IGFBP7]	0.744 (0.597-0.859)	0.686 (0.558-0.796)
[NGAL]•[NephroCheck®]	0.717 (0.569-0.838)	0.664 (0.535-0.777)
